# Efficacy of the in vitro splitting of human preimplantation embryos from ART programs

**DOI:** 10.3906/sag-1710-194

**Published:** 2021-02-26

**Authors:** Marjan OMIDI, Mohammad Ali KHALILI*, Azam AGHA-RAHIMI, Stefania A. NOTTOLA, Fatemeh ANBARI, Azita FARAMARZI, Maria Grazia PALMERINI

**Affiliations:** 1 Research and Clinical Center for Infertility, Yazd Reproductive Sciences Institute,Shahid Sadoughi University of Medical Sciences, Yazd Iran; 2 Department of Reproductive Biology, Shahid Sadoughi University of Medical Sciences, Yazd Iran; 3 Department of Anatomy, Histology, Forensic Medicine, and Orthopedics, Sapienza University of Rome, Rome Italy; 4 Fertility and Infertility Research Center, Kermanshah University of Medical Sciences, Kermanshah Iran; 5 Department of Life, Health, and Environmental Sciences, University of L’Aquila, L’Aquila Italy

**Keywords:** Human embryo, in vitro splitting, blastocyst

## Abstract

**Background/aim:**

The aim of this study was to evaluate the efficiency of in vitro embryo splitting (IES) procedures. We also assessed the quality of the blastocysts developed from embryos obtained from different sources.

**Materials and methods:**

Good quality embryos at 6–8-cell stages were categorized according to their fertilization sources: 1) frozen-warmed donated embryos, 2) chromosomally abnormal embryos, 3) parthenogenetic embryos, and 4) embryos derived from fertilization of in vitro matured oocytes (rescue IVM). After IES, splitting and developmental efficiency was assessed. Furthermore, the quality of the developed blastocysts was evaluated by Hoechst and propidium iodide (PI) staining.

**Results:**

The data showed a high rate of both splitting and developmental efficiency in the frozen-warmed embryos after IES (140% and 71.7%, respectively), followed by chromosomally abnormal embryos (96.8% and 52.5%, respectively). Results of the Hoechst and PI staining showed that the mean ± SD cell numbers of the control group were higher (113.11 ± 16.01) than that of twins A (donor blastomeres embryos, 58 ± 12.2) and B (recipient blastomeres embryos, 50.4 ± 8.5), respectively.

**Conclusion:**

Chromosomally normal embryos enrolled in IES are more potent to develop into viable blastocysts. For research purposes, 1PN and 3PN embryos are the best options for splitting procedures, regardless of the poor quality of developed blastocysts.

## 1. Introduction

Increasing the number of in vitro embryos can be feasible using two techniques: somatic cell nuclear transfer (SCNT) and in vitro embryo splitting (IES). In contrast to cloning by SCNT, which is a completely artificial procedure, IES mimics the natural developmental process that occurs in other embryos created by in vitro fertilization (IVF) techniques (1). In farm animals, pregnancies have been reported by IES that led to the live birth of healthy offspring for sheep (2), horses (3), goats (4), cattle (5), and pigs (6). Moreover, over two decades, IES or embryo twinning has been successfully established in veterinary medicine due to the safe and efficient applications (7). IES efforts in mammals were first achieved in mouse by investigating the developmental potential of twin embryos obtained from cleavage stage embryos at 4- and 8-cell stages (8). Furthermore, split mouse embryos gave rise to healthy monozygotic twins comparable in size and morphology to normal embryos (9). Recently, Tang et al. (10) investigated the developmental potential of single blastomeres isolated from 4-cell mouse embryos. They concluded that the IES could enhance the number of blastocysts; however, the percentage of high quality blastocysts was the same compared to that shown by nonsplit embryos (10). For these approaches, rhesus monkey as a nonhuman primate seems to be the best model close to humans in terms of genetics and physiology (11). However, splitting strategies in rhesus monkeys had limited success in terms of live birth achievements (12,13). One study obtained 368 identical twins of rhesus monkeys produced by splitting, which resulted in four pregnancies and one single live birth from 13 embryo transfers (14). Another study that attempted blastomere separation for 2- and 4-cell stage embryos of rhesus monkeys reported a 33% clinical pregnancy rate with two twin clinical pregnancies, although no live births occurred (15).

In 1993, as a preliminary report, IES was carried out on genetically abnormal (polyploid) human embryos, but the split embryos arrested shortly after a few cell divisions (16). After this report, no further studies were published on human IES until 2008, when scientists obtained a successful development of split human embryos to the blastocyst stage by blastomere biopsy (17). They split 4-cell stage embryos and the four blastomeres obtained were individually cultured in an empty zona pellucida (ZP). Later, Illmensee et al. (18) achieved blastocysts developed from twinned human embryos at the 2- to 5- and 6- to 8-cell stages. To date, very little is known about the quality of the embryos created in such a way and the findings are controversial. It was shown that the blastomeres from late cleavage stage embryos and those derived from good quality embryos had higher splitting efficiency for development to the blastocyst stage (18). On the other hand, a recent study suggested that twin human embryos created by splitting techniques were suitable neither for clinical nor for research purposes (19). It was shown that twin blastocysts from IES were significantly smaller than the control group (17). However, since embryo splitting is a potential method to improve the efficacy of assisted reproductive technology (ART) treatments, this technique was approved by the American Society for Reproductive Medicine’s ethics committee (20).

Regarding the scarcity of human embryos with acceptable quality for research, the application of embryo splitting technology may be a method useful to increase the number of available human embryos. Hence, immature oocytes as well as chromosomally abnormal embryos can be a good source of embryos available for IES application. The aim of the present study was to evaluate the applicable potential of a developed technology, i.e. human IES, regarding its efficacy at different early embryonic stages (splitting efficacy) and success rates of twin embryo development to the blastocyst stage (developmental efficacy). The quality of twinned blastocysts was also assessed regarding cell count and viability. 

## 2. Materials and methods

### 2.1. Samples 

The present study was approved by the ethics committee of the Research and Clinical Center for Infertility, Yazd Reproductive Sciences Institute, Shahid Sadoughi University of Medical Sciences, Yazd, Iran (Reference Number: IR.SSU.MEDICIN.REC.1395.93). Signed forms of consent were obtained from all couples for using discarded oocytes and embryos, and also donated frozen-warmed embryos in our study. Discarded oocytes and embryos consisted of the germinal vesicle (GV) and metaphase I (MI) stage oocytes, and chromosomally abnormal embryos including aneuploid (1PN and 3PN) and parthenogenetic embryos obtained from IVF/ICSI cycles that are usually discarded during ART. The mean ± SD of the patients’ ages in each group was 34.1 ± 4.27 years for frozen-warmed embryos, 33.16 ± 4.22 for aneuploidy embryos, 33.32 ± 4.6 for parthenogenetic embryos, and 32.33 ± 3.6 for embryos created from in vitro matured oocytes. 

### 2.2. IVM, ICSI, and in vitro culture

Retrieved cumulus–oocyte complexes (COCs) were denuded of their cumulus and corona cells using mechanical and enzymatic treatment (80 IU ml–1 hyaluronidase, Irvine Scientific, Irvine, CA, USA) as described previously (21). The denuded oocytes were classified as either mature (MII) or immature (GV or MI) oocytes. Good quality immature oocytes were cultured in blastocyst medium (G-2 v5 Media, Vitrolife, Sweden) (22) supplemented with 75 mIU mL–1 FSH and 75 mIU mL–1 LH (Ferring) at 37 °C in an incubator under 5% CO2 and 95% air with high humidity. The maturity of the oocytes was assessed using a stereomicroscope (Olympus, Tokyo, Japan) 24 h after IVM. The matured oocytes were injected with spermatozoa. Fertilization was assessed 16–22 h after microinjection and the resulting zygotes were cultured in droplets of 20 µL of G-1 v5 Media (Vitrolife, Sweden) until the 8-cell stage.

Regarding the discarded embryos, on fertilization assessment day, the 1PN and 3PN zygotes and unfertilized oocytes were picked out from normally fertilized oocytes and were cultured in droplets of 20 µL of G-1™ v5 media (Vitrolife, Sweden), separately. Finally, grades A and B 8-cell cleaved embryos were selected for in vitro splitting.

### 2.3. Warming procedure

Donated embryos used in the study were cryopreserved using the vitrification method. The embryos were warmed using the RapidWarm Cleave kit (Vitrolife, Sweden) according to the manufacturer’s instructions. Before they were manipulated, the embryos were cultured for at least 4 h in microdrops of 20 µL of G-1 v5 Media (Vitrolife, Sweden) in a humidified atmosphere at 37 °C, in 6% CO2 and 5% O2.

### 2.4. Embryo micromanipulation

**Figure 1:**
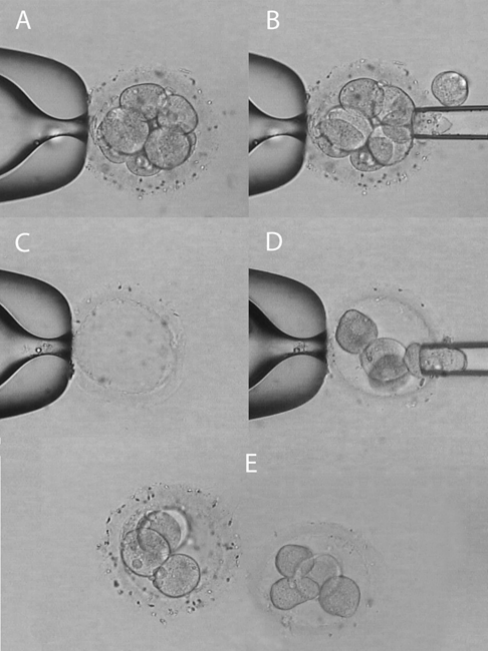
Human embryo twinning procedure. A) A triploid human embryo at cleavage stage with 8 blastomeres was used as the blastomere donor embryo (twin A). B) Half of the blastomeres were biopsied through the created hole in the ZP. C) An empty ZP by source of the discarded oocytes. D) The biopsied blastomeres were inserted into the empty ZP, one by one, as the blastomere recipient embryo (twin B) E) At the end of the procedure, twinned 4-cell embryos were cultured in vitro to the blastocyst stage (magnification 20×).

Prior to biopsy to facilitate separation of blastomeres, the embryos were preincubated in microdrops of Ca+2-Mg+2-free culture medium (PGD medium, Vitrolife, Sweden) overlaid with mineral oil for 10 min at 37 °C. A hole of 35–40 µm was made in the ZP using a 1480-nm wavelength infrared diode laser (OCTAX Laser Shot; MTG, Germany). Arrested embryos or discarded oocytes were evacuated with the same technique to obtain empty ZPs for blastomere recipients. Half of the blastomeres were biopsied by aspiration using a micropipette with inner diameter of 35 µm and inserted one by one into a previously prepared empty ZP (Figure 1). After splitting, donor blastomere embryos (twins A) and recipient blastomere embryos (twins B) were carefully washed and cultured on G-2 v5 Media (Vitrolife, Sweden) covered with mineral oil in 6% CO2 at 37 °C. Development of the twin embryos was followed using an inverted microscope (TE300; Nikon, Tokyo, Japan) every 24 h up to the blastocyst stage.

### 2.5. Blastocyst viability 

For determining the cell number and cell viability, the developed in vitro twin blastocysts were assessed in response to propidium iodide (PI) and bisbenzimide (H33342) fluorochromes, as described earlier (23). Expanded blastocysts were first washed three times in prewarmed Ca+2- and Mg+2-free phosphate buffer saline (PBS-) and then incubated in freshly prepared preincubated staining solutions of Hoechst (H33342, Cat. No. H33342, 5 µg/mL) and PI (Cat. No. P 4127, 300 µg/mL) for 30 and 10 min, respectively, in a humidified atmosphere at 37 °C in 6% CO2 and 5% O2. Blastocysts were then washed three times with warm PBS- to remove the residual dyes and mounted in a drop of glycerol between two lines of paraffin wax. A coverslip was placed on top of the embryos and gently pressed until the embryos were slightly flattened. Prepared samples were examined under a fluorescent inverted microscope (Olympus BX51) at 100× and 400× magnifications using the same excitation wavelength (330–385 nm) and barrier filter (400 nm) to visualize both dyes. H33342 has potency to enter all cells, viable or dead, whereas only cells with altered membrane integrity are permeable to PI. Therefore, the nuclei of viable cells will appear blue, while the dead cells are seen as red. 

### 2.6. Statistical analysis 

Statistical analysis was performed using SPSS (SPSS Inc., Chicago, IL, USA). One-way ANOVA tests, independent samples t-tests, and Mann–Whitney U tests were applied whenever appropriate for comparison of quantitative data between groups. P ˂ 0.05 was considered as statistically significant.

## 3. Results

Embryo twinning was carried out on four categories of human embryos regarding their source, including frozen-warmed donated embryos (n = 20), chromosomally abnormal embryos (n = 32), parthenogenetic embryos (n = 22), and embryos derived from fertilization of in vitro matured oocytes (rescue in vitro maturation (IVM) (n = 18). All of the embryos were at 6–8-cell stage and classified as good quality embryos. The splitting and developmental efficiency of these embryos were evaluated and compared among the four groups. The total mean ± SD of the patients’ ages was 33.24 ± 4.19 years. Moreover, the women’s ages were not different between groups (P = 0.64). The embryos were generated from IVF (n = 29) and ICSI (n = 56) cycles. Embryos created from testicular sperm samples, donation, and assisted fertilization cycles were excluded from the study. 

The data showed a higher rate of both splitting and developmental efficiency in the frozen-warmed embryos (140% and 71.7%, respectively; Table), followed by chromosomally abnormal embryos (96.8% and 52.5%, respectively). The embryos developed after IVM procedures had lower rates of splitting and developmental efficiency (16.6% and 10.7%, respectively; Table). From 28 embryos in this group, only one blastocyst was formed after IES.

**Table:** Table. Comparison of splitting and developmental efficiency* after IES according to the sources of embryos.

Source type of embryos	Embryos manipulated for twinning	Embryos survived after twinning	Embryo development		Splitting efficiency, % (n/N)	Developmental efficiency, % (n/N)
			Morula	Blastocyst		
Frozen-warmed	20	39	10	18	140 (28/20)	71.7 (28/39)
Aneuploid	32	59	13	18	96.8 (31/32)	52.5 (31/59)
Parthenogenetic	22	40	6	2	36.3(8/22)	20 (8/40)
IVM-derived	18	28	2	1	16.6 (3/18)	10.7 (3/28)

*Splitting efficiency was defined as the ratio of morula and blastocysts to embryos manipulated for twinning. Developmental efficiency was defined as the ratio of morula and blastocysts to the embryos available after twinning.

**Figure 2:**
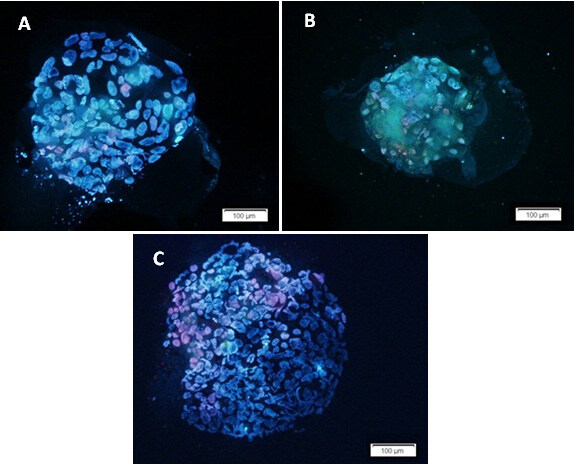
Hoechst and PI staining of the blastocysts as a valuable approach for assessment of the number of viable and dead cells. The figure shows three blastocysts developed from different sources: A) from twin A embryo after in vitro twinning; B) from twin B embryo after in vitro twinning; C) from embryo without any manipulation. The nuclei of viable cells appear blue, while dead cells are seen as red.

**Figure 3:**
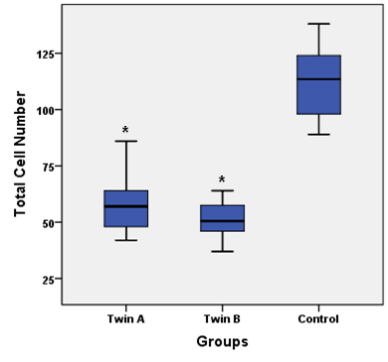
Total cell number of blastocysts compared between groups. There were significant differences between twins A and twins B with control groups (*P < 0.0001). Data are presented as mean ± SD.

**Figure 4:**
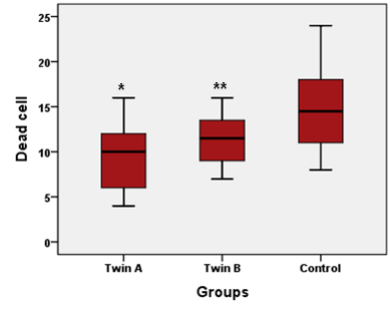
The number of dead cells compared between groups. There were significant differences between twins A and control group and also twins B and control group (*P < 0.0001, **P = 0.04). Data are presented as mean ± SD.

The blastocysts developed from frozen-warmed donated embryos and 3PN zygotes were used for quality evaluation by Hoechst and PI staining. In respect of the total cell number of the blastocysts, our data suggested a significant difference between the split embryo blastocysts (twins A and B) and the blastocysts in the control group (Figures 2 and 3).The mean ± SD cell number of the control group was higher (113.11 ± 16.01) than that of twins A (58 ± 12.2) and B (50.4 ± 8.5) (P ˂ 0.0001). Moreover, the average total number of cells in twins A was insignificantly higher compared to twins B. In subanalysis, it was shown that the number of dead cells differed significantly between the group of twins A and B and the control group, being lower in the split embryo blastocysts (9.38 ± 3.8 in twins A vs. 14.67 ± 4.5 in controls, P ˂ 0.0001; and 11.38 ± 3.1 in twins B vs. 14.67 ± 4.5 in controls, P = 0.04; Figure 4). Furthermore, there was a trend in increasing number of dead cells in twins B compared to twins A, but the differences were not significant (P = 0.27; Figure 4). 

## 4. Discussion

This study documented that IES can be a possible method for increasing the number of viable embryos for any purpose. According to earlier studies, the optimum result would be achieved when high quality embryos at 6- to 8-cell stage are selected for in vitro splitting (18). Despite using high quality embryos for splitting in four groups, our findings showed that embryos with normal fertilization conditions had the best splitting and developmental efficiency compared with abnormal fertilization embryos and embryos following IVM programs (Table). 

In clinical approaches, the numerical increasing of embryos is beneficial for patients enrolled in IVF programs, especially for women at advanced ages and patients considered as “poor responders” (20,24,25). A very limited number of studies have been conducted on in vitro splitting in humans and also in chromosomally normal embryos. The published data, especially from studies on human embryos, are controversial. Several recent studies have suggested that the embryo splitting technique can be a potential method for production of viable and morphologically adequate blastocysts (17,18,24). However, they did not present qualitative assessments of blastocysts created using in vitro splitting. The most recent study by Noli et al. reported that the embryo splitting method by blastomere biopsy was not suitable for clinical aims (19). Our study on donated frozen-warmed embryos with normal fertilization state showed a high splitting efficiency (140%; Table). 

To the best of our knowledge, this is the first study using Hoechst and PI staining for cell numbering and quality assessment of twin blastocysts. Some studies showed a significant difference in size in blastocysts following in vitro embryo splitting regardless of whether they were twins A or B (19), but failed to note the exact total number of derived blastocyst cells. In respect to cell counting, we used the blastocysts developed from frozen-warmed donated embryos and 3PN zygotes. Blastocysts developed from other sources were not stained due to their poor quality. In this regard, the data showed a lower number of cells in twin blastocysts compared to the control ones (Figure 3). This result seems consistent with the fact that the number of blastomeres in twin embryos was reduced in comparison with that of intact embryos. Also, there was a lower number of cells in twins B than in twins A. This result may be due to technical errors. In some in vitro twinning cycles, one blastomere was degenerated during blastomere insertion into the empty ZP, so the number of blastomeres that participated in the blastocyst formation was reduced in recipient embryos. Moreover, the number of dead cells was higher in the control group compared to the other two groups (Figure 4). Our hypothesis is that dead cell number is in positive relation with the total cell number. 

Past studies reported that triploidy can be observed in term-born infants, although survival was limited to a few days (26). It can be concluded from these findings that triploid embryos can be developed to blastocyst stage, even implanted in the endometrium. These chromosomally abnormal embryos discarded after fertilization assessment can thus be a good source of human embryos for surveys of the IES technique. Preliminary studies on human embryo splitting were done using triploid embryos (16,17). Illmensee et al. showed that the micromanipulation of triploid embryos finalized with in vitro splitting did not influence the developmental efficiency of split embryos compared to nonsplit control embryos (17). 

Rescue IVM is a procedure in association with immature oocytes that are retrieved from controlled ovarian hyperstimulation (COH) cycles. Generally, in the routine policy of many IVF settings, these immature oocytes from COH cycles are discarded. However, the study by Veeck et al. (27) showed that these immature oocytes were capable of not only reaching maturation and fertilization in vitro, but also of embryonic development. We thus carried out ICSI following an IVM procedure for immature oocytes and in vitro splitting for deriving good quality embryos subsequently. Nevertheless, our data resulted in the lowest splitting and developmental efficiency for IVM-derived embryos compared to the other embryos (Table). Embryo splitting can have possible benefits in human reproductive research. Due to the scarcity of donated human embryos for research purposes, the splitting technique would be helpful for increasing the number of available viable embryos. In addition, with the preference for blastocyst transfer in ART cycles, there has been a reduction of cleavage stage embryos for research (1). Moreover, embryo splitting provides genetically identical embryos that are a good option for case-control studies. The use of these embryos for comparative research eliminates the probable bias due to the same genetic background. Another usage of embryo splitting is including it in quality control assays, training students and embryologists, and establishing new technologies (19). The main goal of the present study was the evaluation of the splitting efficiency in discarded embryos for these mentioned applications. 

As previously anticipated, a recent report on IES suggested that this new method was suitable neither for clinical use nor for research (19). However, according to the very low rate of 2.3% multiple pregnancies following single embryo transfer cycles compared to the rates of 0.4%–0.45% in natural conception, it seems that IES will find clinical use in reproductive medical science. Regarding the increasing number of embryos created using IES, this procedure can be used for poor responders with low numbers of oocytes or embryos. Accordingly, the assessment of the developmental potential of embryos using IES in poor responders is suggested in future studies. In conclusion, chromosomally normal embryos enrolled in IES are able to develop up to the viable blastocyst stage. Regarding research approaches, abnormal fertilized oocytes (1PN and 3PN) are among the best options for splitting procedures, regardless of the poor quality of developed blastocysts.
